# miRNAs in depression vulnerability and resilience: novel targets for preventive strategies

**DOI:** 10.1007/s00702-019-02048-2

**Published:** 2019-07-26

**Authors:** Nicola Lopizzo, Valentina Zonca, Nadia Cattane, Carmine Maria Pariante, Annamaria Cattaneo

**Affiliations:** 1 Biological Psychiatry Unit, IRCCS Istituto Centro San Giovanni di Dio Fatebenefratelli, Via Pilastroni 4, 25125 Brescia, Italy; 2 Stress, Psychiatry and Immunology Laboratory, Department of Psychological Medicine, Institute of Psychiatry, King’s College London, Coldharbour Lane, London, SE5 9NU UK

**Keywords:** miRNAs, Depression, Stress vulnerability, Stress resilience, Preventive strategies, Antidepressant response

## Abstract

The exposure to stressful experiences during the prenatal period and through the first years of life is known to affect the brain developmental trajectories, leading to an enhanced vulnerability for the development of several psychiatric disorders later in life. However, not all the subjects exposed to the same stressful experience develop a pathologic condition, as some of them, activating coping strategies, become more resilient. The disclosure of mechanisms associated with stress vulnerability or resilience may allow the identification of novel biological processes and potential molecules that, if properly targeted, may prevent susceptibility or potentiate resilience. Over the last years, miRNAs have been proposed as one of the epigenetic mechanisms mediating the long-lasting effects of stress. Accordingly, they are associated with the development of stress vulnerability or resilience-related strategies. Moreover, miRNAs have been proposed as possible biomarkers able to identify subjects at high risk to develop depression and to predict the response to pharmacological treatments. In this review, we aimed to provide an overview of findings from studies in rodents and humans focused on the involvement of miRNAs in the mechanisms of stress response with the final goal to identify distinct sets of miRNAs involved in stress vulnerability or resilience. In addition, we reviewed studies on alterations of miRNAs in the context of depression, showing data on the involvement of miRNAs in the pathogenesis of the disease and in the efficacy of pharmacological treatments, discussing the potential utility of miRNAs as peripheral biomarkers able to predict the treatment response.

## Stress, depression, and miRNAs

It is well known that adverse experiences during the prenatal period or during the first years of life can affect the brain developmental trajectories, leading to an enhanced vulnerability of developing several neurodevelopmental and psychiatric disorders later in life (Lockhart et al. 2018). Indeed, early life stressful experiences represent an important clinical risk factor for the future development of altered behaviours and psychiatric disorders (Cirulli et al. 2009; Fryers and Brugha 2013; Syed and Nemeroff 2017). However, the molecular mechanisms underlying the long-lasting effects of stress are poorly understood, and likely interact with genetic and biological factors throughout the lifespan, a mechanism also known as gene X environment interaction (McEwen 2012; Karatsoreos et al. 2013). In this context, epigenetic mechanisms, such as DNA methylation, DNA hydroxymethylation, histone modification, and miRNAs, have been widely described to mediate the effect of these stressful experiences and to be involved in the vulnerability to depression (Serafini et al. 2014; Januar et al. 2015) as well as for other stress-related mental disorders (Klengel and Binder 2015; Nestler et al. 2016). Indeed, miRNAs act by inducing long-lasting alterations in the expression of key genes and pathways (Hollins and Cairns 2016), that in turn may contribute to the development of the pathology.

MiRNAs, as mentioned before, represent one of the epigenetic mechanisms associated with the long-lasting detrimental effects of stress. MiRNAs are small non-coding RNA molecules (21–24 nucleotides), recognized as one of the fundamental factors involved in the post-transcriptional regulation of gene expression. Indeed, it has been estimated that they are able to modulate up to 60% of protein-coding genes in humans (Catalanotto et al. 2016), generally by repressing the expression of their target mRNAs (Bushati and Cohen 2007; Friedman et al. 2009; Rao et al. 2013). The fine-tuning of gene expression by miRNAs is regulated through the binding to the 3′UTR of mRNA, leading to mRNA degradation or translational repression (Bartel 2004). Also coding domain sequence and 5′UTR are targeted by miRNAs with consequent regulatory effects on gene expression (Helwak et al. 2013; Brummer and Hausser 2014; Catalanotto et al. 2016). Because of their ubiquitous role in gene regulation, they are involved in many physiological processes such as cell proliferation, differentiation, apoptosis, and development. A dysregulation in the miRNAs’ machinery has been related to several pathological conditions, including neuropsychiatric and neurodevelopmental disorders (Ha 2011; Xu et al. 2012; Maffioletti et al. 2014).

MiRNAs have been proposed as non-invasive candidate biomarkers that would allow an early identification of individuals at high risk of developing depression and treatment-resistant patients. Indeed, miRNAs play an important regulatory role in all the biological pathways triggered by stress and they also represent one of the fastest and most dynamic response mechanisms that are able to regulate gene transcription and protein translation in response to stress (Ebert and Sharp 2012).

This review focuses on the relationship between miRNAs, stress vulnerability, or resilience and depression by discussing, both in rodents and in humans, how miRNAs can mediate the long-lasting effects of stress on depression vulnerability or resilience and how changes in miRNAs expression can mediate the response to pharmacological treatments. To this purpose, we searched different combinations of keywords, such as “miRNA”, “stress”, “childhood trauma”, “depression”, “vulnerability”, “resilience”, “mouse”, “rats”, “animal models”, “biomarkers”, “antidepressant”, and “antidepressant response”, using PubMed searching database references. In the following sections, the identified studies are reported in tables and the most important studies will be discussed.

## Effect of stress on miRNAs’ biogenesis

During the last decade, several studies have investigated the exact role of miRNAs, from their production to their role as regulators of gene expression, to understand the key steps that can be affected by stress and thus also involved in the pathogenesis of several psychiatric disorders, especially stress-related disorders, such as depression.

MiRNAs are encoded and transcribed as primary miRNAs (pri–pre-miRNAs) in the nucleus, long primary transcripts with a cap structure at the 5′ end, and polyadenylation at the 3′ end. The subsequent processing into pre-miRNAs starts by the cellular endonuclease Drosha with the microprocessor complex subunit DGCR8/Pasha. There are also evidences suggesting that a minority of miRNAs is processed in a Drosha-DGCR8 independent manner. The pre-miRNA is cleaved by the endoribonuclease Dicer in cytoplasm, producing a short double-stranded miRNA duplex, which is then processed in a mature miRNA. Subsequently, it is incorporated in the RNA-induced silencing complex, constituted by the components of the Argonaute family protein (Wahid et al. 2010; Nakanishi 2016). β-catenin protein plays a key protective role in stress conditions and in the upstream miRNA synthesis, through the control of the enzyme Dicer1 according to a recent study conducted by Dias et al. (2014), and indeed, the presence of lower levels of Dicer1, as a consequence of stress exposure, has been also associated with an enhanced vulnerability to stress (Lim et al. 2011; Mori et al. 2012; Dias et al. 2014).

For example, Wingo et al. (2015) investigated the impact of Dicer1 regulation in patients with post-traumatic stress disorder and with comorbid depression, by reporting reduced levels of Dicer1 in patients as compared to controls; this effect was replicated in other two independent cohorts (Wingo et al. 2015). Interestingly, altered levels of several proteins involved in β-catenin activity control, such as the enzyme Glycogen synthase kinase 3, have been shown in depressed patients (Jope and Roh 2006; O’Brien and Klein 2007). Similarly, He et al. (2012) reported an association between genes involved in miRNAs’ biogenesis and depression. Indeed, they investigated single-nuclear polymorphisms (SNPs) within a wide panel of genes including DiGeorge syndrome chromosomal region 8 (DGCR8), Argonaute1 (AGO1), and Gem-Associated Protein4 in 314 depressed patients and 252 healthy controls, showing that SNPs within DGCR8 (rs3757) and AGO1 (rs636832) are associated with an increased risk for depression (He et al. 2012).

## Role of miRNAs in stress response and neuropathological conditions in humans

The activation or the inhibition of gene transcription is often coordinated by the action of several miRNAs that act in clusters (Catalanotto et al. 2016). Although many genes are responsive to a single miRNA and an individual gene can be targeted by several miRNAs, the ideal strategy would be to study the coordinated action of all miRNAs and their target genes as well as the direction of their modulation and to analyse the pathways and the complex networks in which they are involved (Carroll et al. 2013).

Only few studies have focused the attention on changes in miRNAs levels in association with stress exposure or depression in humans, and in this manuscript, we discussed those conducted in post-mortem brains, in peripheral blood and in sperm samples. In Table 1, we reported the studies which showed alterations in miRNAs levels in depressed patients or in healthy subjects exposed to stressful experiences. We used as input keywords for PubMed “miRNAs”, “Stress”, “Childhood Trauma”, “Depression”, “Resilience”, and “Vulnerability”.

**Table 1:** Summary of the studies showing alterations in miRNAs levels in depressed patients or in control subjects exposed to stress

microRNA/Gene	Methods	Principal findings	References
miR-449a, miR-34c	Microarray analysis and real-time PCR	miR-34/449 dysregulation in sperm of subjects exposed to trauma	Dickson et al. (2018)
miR-124-3p	qPCR-based transcript quantification	Significant increase in the expression of miR-124-3p in post-mortem brains of depressed patients versus control subjects	Roy et al. (2017)
miR-218, DCC	Real-time PCR and DCC immunofluorescence	miR-218 as associated with susceptibility to stress-related disorders	Torres-Berrio et al. (2017)
miR-15a	Real-time PCR and NGS sequencing (MiSeq)	Increased levels of miR-15a in blood of subjects exposed to childhood trauma or to the oral administration of Dexamethasone	Volk et al. (2016)
Let-7b, let-7c	Real-time quantitative reverse-transcription PCR	Decreased Let-7b and let-7c baseline expression levels in treatment-resistant depressed patients as compared to controls	Gururajan et al. (2016)
DICER1	Genome-wide differential gene expression profiles	Role of DICER1 in the mechanisms underlying PTSD and Depression	Wingo et al. (2015)
miR-511, GDNF	Quantitative real-time PCR, miRNA transfections, immunoblotting and immunochemistry	miRNA targeting isoform-specific GFRα1 receptor in shaping neuronal responses to the neurotrophin GDNF	Maheu et al. (2015)
miR-144-5p	Real-time PCR	Reduced plasma miR-144-5p levels in depressed patients as compared to controls	Wang et al. (2015)
miR-16	Real-time quantitative reverse-transcription PCR	Decreased CSF miR-16 levels in drug-free depressed patients compared to control subjects	Song et al. (2015)
Let-7 family	Polymerase chain reaction–restriction fragment length polymorphism and DNA sequencing assays	Association between rs10877887 and rs13293512 polymorphisms within let-7 family and an increase risk for depression	Liang et al. (2015)
DICER1	Genome-wide β-catenin enrichment mapping	β-catenin protective role through miRNA production	Dias et al. (2014)
miR-135a	Western blot and real-time PCR	Reduced blood miR135a levels in depressed patients versus controls	Issler et al. (2014)
miR-26b, miR-1972, miR-4485, miR-4498, miR-4743	Real-time quantitative reverse-transcription PCR	Up-regulated levels of 5 miRNAs in depressed patients compared to healthy controls	Fan et al. (2014)
miR-132, miR-182	Real-time PCR, ELISA	Role of miR-132 and miR-182 in BDNF regulation	Li et al. (2013)
DGCR8, AGO1 and GEMIN4	High-resolution melting and Real-Time PCR	DGCR8 rs3757 and AGO1 rs636832 as associated with an increased risk for depression	He et al. (2012)
Panel of 21 miRNAs	Real-time quantitative reverse-transcription PCR	Down-regulation in miRNA expression in depressed suicide subjects versus control subjects	Smalheiser et al. (2012)

### Studies on post-mortem brains

Most of the studies in the literature are focused on the possible role of miRNAs in driving the effect of stress exposure on depression vulnerability. For example, Smalheiser et al. (2012) investigated miRNAs network in post-mortem brains of antidepressant-free depressed suicide patients using multiplex RT-PCR, and they have identified a network of miRNAs able to target several genes, which have been already demonstrated to be involved in depression vulnerability. In particular, they found that miRNAs expression was overall significantly down-regulated in cases versus controls, with a particular effect on 21 miRNAs, which have a common seed region (Smalheiser et al. 2012). Among the predicted targets of these 21 altered miRNAs, they found estrogen receptor alpha (ESR1), which is targeted by 3 different down-regulated miRNAs (miR-148b, miR-301a, and miR-496). Interestingly, ESR1 has been recently associated with resilience to depression (Lorsch et al. 2018). Moreover, among all the validated target genes of the 21 modulated miRNAs, they also found the DNA Methyltransferase 3 Beta (targeted by miR-148b), the Vascular endothelial growth factor A (VEGF-A) (targeted by miR-20b, miR-20a, miR-34a, and miR-35b-5p), and the B-cell lymphoma 2 (targeted by miR-34a) genes, that interestingly have all been already associated with depression vulnerability (Smalheiser et al. 2012).

Maheu et al. (2015) analysed whether (and which) miRNAs can alter the neuronal response to Glial Cell Line-Derived Neurotrophic Factor (GDNF), which was previously related to the development of coping stress abilities and to the neuroprotective neuroplasticity-related mechanisms, and was also found reduced in depressed patients (Uchida et al. 2011; Lin and Tseng 2015). In particular, the authors showed an inhibitory action of the miR-511 on the GDNF receptor expression (GFRα1a and GFRα1b) in depressed suicidal subjects’ post-mortem brains (Maheu et al. 2015). Their results showed that miR-511 inhibition occurs specifically on the GFRα1a receptor. Altered GFRα1a-to-GFRα1b ratio is associated with greatly enhanced mitogen-activated protein kinase (MAPK) signalling, highlighting the importance of isoform-specific GFRα1 receptor regulation in shaping neuronal responses to this neurotrophin.

In addition, miR-135a and miR-124-3p have been investigated as possibly involved in depression vulnerability (Issler et al. 2014; Roy et al. 2017). Issler et al. (2014) reported lower levels of miR-135a in the raphe nuclei of suicide victims as compared to control subjects and the same results were also found at peripheral level (in the whole blood); in parallel, they demonstrated alterations in miR-135a target genes involved in the regulation of Serotonin (5-HT) neuron-related genes such as the 5-HT transporter, SLC6A4, and the 5-HT receptor, HTR1A, suggesting a role for miR-135a as a potential blood biomarker for depression vulnerability (Issler et al. 2014). Another miRNA that has been investigated is the miR-124-3p, which Roy et al. (2017) found increased in the Pre-Frontal Cortex of post-mortem brains of depressed patients as compared to controls. They also found reduced expression of the miR-124-3p target genes: Glutamate receptor 3, Glutamate receptor 4, and Glucocorticoid receptor (NR3C1), which are involved in stress response and neuroplasticity (Roy et al. 2017).

MiR-218, which targets Netrin-1 guidance cue receptor Deleted in Colorectal Cancer (DCC), has been proposed as a new epigenetic marker of stress vulnerability. Indeed, alterations in DCC expression appear to be involved in resilience or susceptibility to psychiatric disorders involving prefrontal cortex dysfunction. Briefly, Torres-Berrio et al. (2017) investigated the mechanism of co-expression of DCC and miR-218 in pyramidal neurons of human prefrontal cortex (Flores 2011; Manitt et al. 2013). The expression of miR-218 and DCC was quantified in post-mortem prefrontal cortex tissue samples obtained from antidepressant-free depressed subjects who committed suicide and sudden-death controls, using Real-Time PCR and immunofluorescence; increased DCC and reduced miR-218 expression levels were observed in the prefrontal cortex of depressed subjects in line with the expected inverse relationship between the miRNAs and the mRNA levels of the target gene. Therefore, this imbalance between miR-218 and its target DCC levels might be a key mechanism of susceptibility to stress (Torres-Berrio et al. 2017).

### Studies on peripheral blood, cerebrospinal fluid, and sperm samples

As compared to the studies conducted in post-mortem brains, much more evidences are available on peripheral blood from control and depressed subjects who experienced stressful events.

One of the first studies has been published by Fan et al. (2014), who identified changes in 26 miRNAs in peripheral blood mononuclear cells obtained from three depressed patients compared to three control subjects using miRNAs’ microarray expression profiling. The authors validated the microarray results using Real-Time PCR in an enlarged cohort of 91 depressed patients and 46 control subjects matched for age and gender. All the 26 miRNAs were up-regulated in depressed patients except for miR-338 and, among these 26 miRNAs, miR-26b, miR-1972, miR-4485, miR-4498, and miR-4743 showed statistical significance. Investigating more in detail the pathways in which those miRNAs are involved, the authors observed a particular enrichment in pathways related to the axon guidance, glutamatergic synapse, Wnt signalling, ErbB signalling, mTOR signalling, VEGF signalling, and long-term potentiation. Those findings are in line with the literature data that suggest a possible involvement of these systems in the biological mechanisms related to depression development (Voleti and Duman 2012; Fan et al. 2014; Abelaira et al. 2014).

MiRNAs have been also investigated as possible mechanisms underlying depressive development. For example, Wang et al. (2015) showed that miR-144-5p baseline levels were reduced in depressed patients as compared to controls and were inversely correlated with Montgomery-Asberg Depression Rating Scale (MADRS) scores, suggesting miR-144-5p as a possible biomarker associated with depression severity (Wang et al. 2015).

Another miRNA suggested to be implicated in the physiopathology of depression is miR-16, as it acts in the modulation of serotonin transmission in the brain (Song et al. 2015). In particular, miR-16 levels measured in the CSF from depressed patients were significantly lower than those in controls; moreover, CSF miR-16 was negatively correlated with Hamilton depression rating scale scores and positively associated with CSF serotonin levels, suggesting this miRNA as a promising biomarker for mood changes (Song et al. 2015).

After an exploratory analysis using online miRNA analysis software *Targetscan*, Li et al. (2013) focused their attention on miR-132 and miR-182, which target the Brain-Derived Neurotrophic Factor (BDNF) gene. BDNF plays an essential role in neurodevelopment and neuroplasticity and its levels have been found reduced both at peripheral and central levels in depressed patients as compared to controls in several studies (Cattaneo et al. 2010; Aas et al. 2018; Nunes et al. 2018). Li et al. (2013) measured serum BDNF, miR-132, and miR-182 mRNA levels in 40 depressed patients versus 40 healthy controls, reporting lower BDNF levels in association with depression, whereas the levels of miR-132 and miR-182 were significantly increased (Li et al. 2013). A reduction in BDNF protein levels has been suggested to promote the development of depression and its treatment resistance (Lee and Kim 2010; Ihara et al. 2016).

A recent work of Gururajan et al. (2016) showed that let-7b and let-7c can regulate the expression of 27 genes in the PI3 K-AKT-mTOR signalling pathway, which has previously been reported to be deregulated in depression (Jernigan et al. 2011; Chandran et al. 2013). Indeed, the expression of the miRNAs let-7b and let-7c was found significantly decreased in a group of treatment-resistant depressive patients compared with control subjects (Gururajan et al. 2016).

The impact of miRNAs on depression vulnerability has been also evaluated from a genetic point of view. Liang et al. (2015) have investigated the possible contribution of several SNPs located within the promoter of let-7 family in the mechanisms underlying depression vulnerability by genotyping 237 depressed patients and 296 healthy controls. The let-7 family has been the first to be discovered in humans with well-known roles in neurogenesis and synapse formation (Rehfeld et al. 2015) and a possible involvement in depression development (Maffioletti et al. 2016). The authors here showed that both the rs10877887CC and the rs13293512CC let-7 genotypes increased the risk for depression onset (Liang et al. 2015).

Only a few studies have investigated the possible effects of stressful life events on miRNAs modulation in humans. Among them, Volk et al. (2016) suggested an important role for miR-15a in the development of coping strategies versus stressful exposures. Indeed, they analysed miR-15a expression levels in RNA samples from peripheral blood cells of young healthy male subjects following an oral administration of the glucocorticoid receptor agonist dexamethasone. They reported a significant up-regulation of miR-15a at 3- and 6-h post-treatment, an effect that interestingly was also present in peripheral blood cells of control subjects exposed to childhood trauma when compared to non-exposed individuals. Given the previous evidence of the key role of the Hypothalamic–Pituitary–Adrenal (HPA) axis and of childhood trauma in the development of depression (Juruena 2014; Keller et al. 2017; Juruena et al. 2018), the authors’ findings indicate that miR-15a is potentially regulated by the activation of the stress hormone system in humans and it is involved in stress vulnerability as well as in the onset of depression (Volk et al. 2016).

Recently, Dickson et al. (2018) have investigated also the effects of childhood trauma on miRNAs levels using a different biological sample, the sperm of voluntary healthy subjects. To screen and identify candidate miRNAs whose expression in sperm correlates with Adverse Childhood Experience (ACE) score, they selected sperm samples from five men with the highest ACE scores (≥ 4) and five with the lowest (0–1) and performed miRNAs’ array analysis. Multiple members of the miR-34/449 family, in particular miR-449a and miR-34c, showed pronounced reduction in the subjects with highest ACE score (Dickson et al. 2018).

Therefore, from a revision of the literature, up to now, most of the studies in humans considered only partially the role of miRNAs in the stress vulnerability and stress resilience mechanisms as studies have looked mainly at miRNAs and their targeting genes levels in depressed patients versus controls, taking into account only partially the impact of effects of stressful life events and the different individual reactions.

## Role of miRNAs in the mechanisms underlying stress vulnerability and stress resilience

Albeit stress is an important risk factor for the development of several psychiatric diseases, its response and impact on each individual can be different, as it is not only attributable to the exposure to stressful events through lifetime, rather to a plethora of risk factors, including the individual genetic background, the environment, and how these factors can interact with each other, through a mechanism also known as Gene X Environment (GXE) interaction.

Based on this, although traumatic life events (especially those occurring early in life or during adolescence) represent an important clinical risk factor for depression vulnerability, not all the exposed individuals develop stress-related psychiatric disorders, as, contrarily, they acquire coping strategies and become resilient (Wu et al. 2013; Pfau and Russo 2015). For this reason, the identification of molecular mechanisms and biomarkers associated with vulnerability or resilience will help in the identification of subjects at high risk; monitoring and preventive programmes could be then proposed to the identified high-risk individuals to minimize their susceptibility for the development of psychopathology later in life.

On the other hand, the identification of mechanisms underlying stress resilience might be useful for the identification of biological processes that confer “protection” versus stress exposures, which thus will be useful for the discovery of novel targets for the development of new drugs and preventive strategies.

However, up to now, most of the studies reported in literature have analysed the effect of stress without focusing on potential differences in relation to stress vulnerability or resilience. Accordingly, in this paragraph, we will show the main findings regarding the effect of stress on miRNAs levels (Table 2), mentioning also the latest research articles on distinct miRNAs changes in relation to stress vulnerability and stress resilience (Table 3) in animal models. To do this, we searched in PubMed studies using the keywords “stress” and “miRNAs”, and we included only findings related to the effect of stress on miRNAs modulation. Within the 12 relevant studies, all reported in Table 2, in the present paragraph, we will deeply discuss only those that are focused on miR-124 and miR-16, as they are the most investigated miRNAs in the context of stress.

**Table 2:** List of studies reporting miRNAs modulated by stress exposure

miRNAs modulated by stress exposure		
miRNA	Stressful paradigm	References
miR-134	Chronic unpredictable mild stress	Yu et al. (2018)
miR-9-5p miR-128-1-5p miR-382-5p miR-16-5p miR-129-5p miR-219a-5p	Chronic mild stress	Buran et al. (2017)
miR-135a miR-16	Inescapable stress	Liu et al. (2017)
miR-17-92		Jin et al. (2016)
Several known miRNAs	Chronic unpredictable mild stress	Ma et al. (2016)
miR-383 miR-764	Chronic unpredictable mild stress	Duan et al. (2016)
16 miRNAs	Electro-acupuncture	Duan et al. (2016)
miR-326	Chronic variable mild stress	Aschrafi et al. (2016)
miR-10B	Chronic unpredictable stress Sleep deprivation	Jiang and Zhu (2015)
miR-124	Chronic social defeat Chronic unpredictable mild stress	Bahi et al. (2014) Cao et al. (2013)
miR-16	Maternal deprivation and unpredictable stress	Bai et al. (2012)
miR-34c	Acute restraint stress and chronic social defeat stress	Haramati et al. (2011)

**Table 3:** List of studies reporting involvement of miRNAs in the mechanisms associated with resilience and/or vulnerability to stress

miRNAs involved in stress resilience and vulnerability		
miRNA	Stressful paradigm	References
miR-455-3p miR-30e-3p	Chronic social defeat	Pearson-Leary et al. (2017)
miR-124	Chronic unpredictable mild stress Chronic ultra-mild stress	Xu et al. (2017) Higuchi et al. (2016)
miR-16	Chronic mild stress	Zurawek et al. (2016)
miR-24-2-5p miR-27a-3p miR-30e-5p miR-362-3p miR-139-5p miR-28-3p miR-326-3p miR-99b-5p	Chronic social defeat	Chen et al. (2015)
miR-9 miR-326	Maternal deprivation Chronic unpredictable stress	Zhang et al. (2015)
miR-504	Maternal deprivation	Zhang et al. (2013)

Bahi and colleagues showed alterations in miR-124a levels in rats exposed to chronic social defeat stress, which consists of placing animals in the home cage of an unfamiliar rat and allowing them to interact until the intruder shows a defeat position for approximately 3 s. The authors reported higher levels of miR-124a in the hippocampus of stressed rats, whereas no differences were observed in the prefrontal cortex as compared to matched controls (Bahi et al. 2014). Interestingly, miR-124 directly targets and modulates the expression of BDNF, which is known to be involved in neurogenesis and it is also involved in the pathophysiology of depression (Castren et al. 2007; D’Sa et al. 2002; Duman and Monteggia 2006). In line with the presence of an up-regulation of miR-124a, Bahi and colleagues also found reduced BDNF levels in the hippocampus of the same animals, supporting that BDNF gene expression changes are in part associated with changes in miR-124a levels and suggesting that miR-124a might participate in the induction of depressive-like behaviour through a direct regulation of BDNF gene expression (Bahi et al. 2014).

Also miR-16 has been suggested to participate in the mechanisms underlying stress response through the regulation of one of its predicted target genes, BDNF (Bai et al. 2012). It has been shown that rats exposed to maternal deprivation (MD) exhibited a depressive-like behaviour which paralleled a down-regulation of BDNF mRNA and protein levels along with an up-regulation of miR-16 expression levels, an association that was supported by a significant negative Pearson correlation (Bai et al. 2012).

To discuss studies on miRNAs expression in relation to stress vulnerability and resilience, we searched in PubMed for animal studies using the keywords “stress”, “miRNAs”, “vulnerability”, and “resilience”, and we found eight relevant studies which have been all reported in Table 3.

In 2015, Chen and collaborators were one of the first groups looking at vulnerability and resilience as different and distinct responses to stress. In their study, animals were behaviourally tested and divided into the vulnerable or resilient groups, depending on the delay latencies which they used to display the defeat position: rats showing an average of latency score < 300 s were classified as short latency rats and these animals represented the vulnerable group. On the other hand, the resilient group was represented by the long latency rats, which displayed a latency score > 300 s. MiRNAs profiling on blood collected before the stress exposure showed four miRNAs significantly decreased in rats that developed a vulnerable phenotype: miR-24-2-5p, miR-27a-3p, miR-30e-5p, and miR-362-3p. Conversely, the expression levels of another panel of miRNAs (miR-139-5p, miR-28-3p, miR-326-3p, and miR-99b-5p) were reduced in animals showing a resilient phenotype (Chen et al. 2015).

Whether a few studies analysed the entire profile of miRNAs using -omics approaches, others focused the attention on single and specific candidate miRNAs, such as miR-124 and miR-16 (Xu et al. 2017; Higuchi et al. 2016; Zurawek et al. 2016). For example, Xu and collaborators reported that rats exposed to a chronic unpredictable mild stress or treated with dexamethasone during adolescence showed an up-regulation of miR-124 expression levels in the basolateral amygdala as compared to non-stressed animals (Xu et al. 2017).

The same miRNA was found reduced in the hippocampus of mice exposed to chronic ultra-mild stress (1-week period of repeated mild stressful events), which developed depression-like behaviour, whereas the tricyclic antidepressant (TCA) imipramine reverted the effect of stress (Higuchi et al. 2016). In the same study, an over-expression of the viral-mediated miR-124 in murine hippocampal neurons conferred behavioural resilience to chronic mild unpredictable stress, whereas the infusion of the anti-miR-124 was able to drive enhanced vulnerability to stress, supporting that a modulation of this miRNA can contribute to stress resilience (in case of over-expression) or vulnerability (in case of down-regulation). However, these results are in contrast with the presence of increased expression levels of miR-124 in stressed animals reported in the previously described studies (Xu et al. 2017; Bahi et al. 2014; Cao et al. 2013). These discrepancies might be due to different animal species, to differences in the stress paradigm, and to the interactions of these factors. For this reason, further studies are required to better clarify the role of these miRNAs in determining stress vulnerability or resilience and which are the target genes and pathways involved.

MiR-16 has also been investigated as a key regulator of stress response. It has been shown, for example, that it is an actor in controlling hippocampal neurogenesis and in regulating serotonin transporter (SERT) levels after fluoxetine treatment (Baudry et al. 2010; Launay et al. 2011). In particular, Zurawek and colleagues showed an up-regulation of miR-16 expression levels in serum samples of resilient rats after 1 week of chronic mild stress paradigm as compared both to animals that developed an anhedonic-like phenotype and also to control animals (Zurawek et al. 2016). Furthermore, after 7 weeks of chronic mild stress paradigm, anhedonic-like animals showed a significantly down-regulation of miR-16 in the ventral tegmental area (VTA) and in the hippocampus as compared to control and resilient rats.

Besides miR-124 and miR-16, other miRNAs have been associated with resilience and/or vulnerability to stress, such as miR-9, miR-326, and miR-504. Zhang and colleagues investigated the role of miRNAs as possible mediators of the long-lasting effect of early life stress exposures on enhanced susceptibility to chronic stress in adulthood. Rats were exposed to maternal deprivation during the first 14 days of life and then daily exposed to chronic unpredictable stress from week 10; a group of animals was also treated with escitalopram from week 14 with a daily 4-weeks intraperitoneally infusion. MiR-9 was found to be down-regulated in the striatum of rats exposed to both maternal deprivation with or without exposure to chronic mild stress, whereas miR-326 levels were down-regulated only in rats exposed to chronic mild stress, suggesting that only miR-326 is sensitive to stress in adulthood. Furthermore, escitalopram was not able to normalize the decrease in miR-9 levels, whereas miR-326 levels were increased by 4 weeks of treatment. The authors suggested that maternal deprivation enhanced the development of depression-like behaviours and increased resistance to escitalopram treatment in rats by promoting a down-regulation of miR-9, whereas miR-326 could represent a new target for the development of novel antidepressant drugs (Zhang et al. 2015). This study is in line with previous findings that showed the presence of an up-regulation of miR-504 which paralleled a down-regulation of dopamine receptor 2 (DRD2) in rats’ nucleus accumbens, in association with enhanced vulnerability to stress in adulthood in rats previously exposed to maternal deprivation (Zhang et al. 2013).

Finally, Pearson-Leary and colleagues identified miR-455-3p and miR-30e-3p as involved in the development of stress-coping strategies in rats exposed to chronic social defeat stress. The rats were exposed to social defeat for 7 days and then behaviourally tested to identify vulnerable or resilient animals; interestingly, increased miR-455-3p levels were observed only in the ventral hippocampus (vHPC) of resilient mice, whereas an increase in miR-30e-3p levels was observed specifically in vulnerable mice. Pathway analyses identified inflammatory and vascular remodelling pathways as enriched by genes targeted by these miRNAs, suggesting that inflammatory processes and vascular remodelling in the vHPC might be directly involved in the development of stress vulnerability (Pearson-Leary et al. 2017).

## Antidepressant drugs and miRNAs

The guidelines for the treatment of depression suggest as the first-line treatment the selective serotonin reuptake inhibitors (SSRIs), the serotonin noradrenaline reuptake inhibitors (SNRIs), and other drugs including agomelatine, bupropion, mirtazapine, and vortioxetine (Mora et al. 2018; Kennedy et al. 2016). However, approximately a third of treated depressed patients report low or absent symptomatology improvement, ending up with a drop out of the therapy, or leading to unhealthy consequences, such as the worsening of symptomatology, resulting also in suicide ideation.

Giving the possible role of miRNAs in the pathogenesis of depression, a growing body of evidence has also suggested that changes in their expression may participate in the efficacy of antidepressant treatment and thus could represent useful peripheral markers to monitor treatment response.

In the following paragraphs, we will review studies that investigated possible changes in miRNAs’ expression in association with antidepressant treatment response. A summary of the studies on miRNAs and treatment response in humans are provided in Table 4, whereas those in animal models are shown in Table 5.

**Table 4:** Summary of miRNAs modulated by antidepressant drugs in human samples

miRNAs	Samples/tissue	Antidepressant drug	Results	References
miR-16 miR-30 miR-34 miR-128 miR-132 miR-134 miR-182 miR-183 miR-185 miR-212	Serum	Different SSRI and SNRI (4 weeks of treatment)	In patients treated with SSRI, miR-16, miR-183, and miR-212 levels increased significantly after 4 weeks of antidepressant treatment	Lin et al. (2018)
miR-451a miR-34a-5p miR-221-3p	Serum	Paroxetine (8 weeks of treatment)	Depressed patients had lower serum miRNA-451a, and higher serum miRNA-34a-5p and miRNA-221-3p as compared to controls; miRNA-34a-5p and miRNA-221-3p decreased, whereas miRNA-451a increased after paroxetine treatment	Kuang et al. (2018)
miR-151a-3p miR-221/222	In silico study	SSRI (paroxetine)	miR-151a-3p, miR-221/222, and their respective target genes, CHL1 and ITGB3, are implicated in the response to SSRI	Oved et al. (2017)
miR-130b miR-26a/26b let-7f miR-770-5p miR-34c-5p	Human U87 glioblastoma cells	Escitalopram (24, 48, 72 h)	Significant increase in let-7f, after both 48 h and 72 h and of miR-26a after 48 h of treatment	Maffioletti et al. (2017)
miR-146a-5p miR-146b-5p miR-425-3p miR-24-3p	Blood cells	Duloxetine (8 weeks of treatment)	Differential expression of miR-146a-5p, miR-146b-5p, miR-425-3p, and miR-24-3p according to treatment response	Lopez et al. (2017)
miR-1202 miR-135a miR-16	Blood cells	Escitalopram or Desvenlafaxine or Duloxetine (8 weeks of treatment)	In two different cohorts, responders displayed lower baseline miR-1202 levels compared with non-responders; miR-1202 levels increased after 8 weeks of antidepressant treatment	Fiori et al. (2017)
miR-1202	Blood cells	Desvenlafaxine (8 weeks of treatment)	Changes in peripheral miR-1202 levels were associated with changes in brain regions associated with depression and antidepressant response	Lopez et al. (2017)
miR-572 miR-663a	Neuroblastoma cell lines	Fluoxetine (24 h)	Fluoxetine increased the expression of both miR-572 and miR-663a	Mundalil Vasu et al. (2016)
414 miRNAs	Blood cells and PBMC	Citalopram (8 weeks of treatment)	414 miRNAs modulated highly correlated set of genes (modules) which are associated with clinical improvements.	Belzeaux et al. (2016)
222 miRNAs	RNA from plasma	Escitalopram (12 weeks of treatment)	Of 222 miRNAs, 40 miRNAs were differently expressed after treatment; 23 significantly over-expressed and 17 down-regulated	Enatescu et al. (2016)
miR-124	PBMC	Different antidepressant drugs (8 weeks of treatment)	The expression level of miR-124 was significantly down-regulated after antidepressant drug treatment	He et al. (2016)
miR-355	Blood cells	Citalopram (4 weeks of treatment)	Citalopram up-regulated miR-335 expression and down-regulated GRM4 mRNA levels	Li et al. (2015)
miR-135	Blood cells	Escitalopram (12 weeks of treatment)	Trend for higher expression after CBT vs escitalopram	Issler et al. (2014)
miR-1202	Human Neural Progenitor cells (NPCs) and blood cells	Citalopram or Imipramine (24 h or 15 days for NPCs; 8 weeks of treatment for depressed subjects)	MiR-1202 was up-regulated in NPCs after acute treatment with both citalopram and imipramine. Moreover, remitter depressed patients showed an up-regulation of miR-1202 after citalopram treatment	Lopez et al. (2014)
miR-221 miR-222	Human Lymphoblastoid cell lines	Paroxetine (21 days of treatment)	Decreased expression of miR-221 and miR-222 after paroxetine treatment	Oved et al. (2013)
30 miRNAs	Blood cells	Escitalopram (12 weeks of treatment)	Identification of 30 miRNAs modulated by antidepressant treatment: 28 miRNAs were up-regulated, and 2 miRNAs were down-regulated	Bocchio-Chiavetto et al. (2013)
42 miRNAs	Human Lymphoblastoid cell lines	Paroxetine (3 days of treatment)	Of the 42 miRNAs, 19 had higher expression levels in the LCLs with high sensitivity to paroxetine	Oved et al. (2012)
miR-145 miR-20b	PBMC	Different antidepressant drugs (8 weeks of treatment)	Increased miRNAs levels during treatment only in responder patients	Belzeaux et al. (2012)

**Table 5:** Summary of miRNAs modulated by antidepressant drugs in animal models

miRNAs	Samples/tissue	Antidepressant drug	Results	References
miR-134	Basolateral amygdala (rat)	Ginsenoside Rg1 (5 weeks of treatment)	Ginsenoside Rg1 induced an up-regulation of miR-134 in basolateral amygdala of rats exposed to chronic unpredictable mild stress	Yu et al. (2018)
626 miRNAs	Hippocampus (mice)	Paroxetine (2 weeks of treatment)	64 miRNAs showed significant changes after drug exposure	Miao et al. (2018)
miR-448-3p miR-764-5p miR-1264-3p miR-1298-5p miR-1912-3p	Hippocampus (mice)	Ketamine (single infusion)	Treatment with ketamine significantly increased the levels of miR-448-3p, miR-764-5p, miR-1264-3p, miR-1298-5p, and miR-1912-3p after 24 h	Grieco et al. (2017)
miR-124	Hippocampus (mice)	Imipramine (continuous infusion for 2 or 4 weeks)	Imipramine reversed the effects on the down-regulation on miR-124 induced by chronic unpredictable mild stress	Higuchi et al. (2016)
miR-132 miR-18a miR-134 miR-124a	Frontal lobe and hippocampus (mice)	Duloxetine (3 weeks of treatment)	MiR-132 and miR-18a were up-regulated, whereas miR-134 and miR-124a were down-regulated after duloxetine administration in the hippocampus. In the frontal lobe, duloxetine induced the up-regulation of miR-18a compared to control animals	Pan and Liu (2015)
miR-9 miR-326	Brain tissue (rat)	Escitalopram (4 weeks of treatment)	Escitalopram did not change the expression of miR-9, whereas the aberrant levels of miR-326 were normalized by the antidepressant drug	Zhang et al. (2015)
miR-135	Raphe Nuclei (mice)	Imipramine or Fluoxetine or Reboxetine (24 h-acute; from 18 to 21 days of treatment-chronic)	Chronic and acute treatment with imipramine or fluoxetine was able to up-regulate miR-135 in raphe nuclei	Issler et al. (2014)
miR-132	Hippocampus (male mice)	Oleanolic acid (3 weeks of treatment)	Oleanolic acid induced the up-regulation of miR-132	Yi et al. (2014)
15 miRNAs	Hippocampus (mice)	7-Chlorokynurenic acid (7-CTKA) (intraperitoneal injection)	7-CTKA exerted a rapid antidepressant effect by modulating 15 hippocampal miRNAs involved in the TrkB-ERK/Akt pathways	Liu et al. (2015)
miR-206	Hippocampus (rat)	Ketamine (3 days of treatment)	MiR-206 was down-regulated after ketamine administration compared to control animals	Yang et al. 2014
miR-125 miR-182	Hippocampus (rat)	Chaihu Shugan San	MiR-125 and miR-182 recovered to normal levels after intervention with Chaihu Shugan San	Cao et al. (2013)
miR-1971	Prefrontal cortices (PFCs)	Fluoxetine (28 days of treatment)	Fluoxetine in shocked mice was associated with a significant reduction in miR-1971 expression	Schmidt et al. (2013)
miR-212	Dentate gyrus and whole blood (rat)	Electroconvulsive stimulation (ECS) (from one-acute to ten-chronic stimulations)	MiRNA miR-212 were significantly increased in rat dentate gyrus following both acute and chronic ECS. MiR-212 levels increased in whole blood only after chronic ECS	Ryan et al. (2013)
miR-598-5p	Hippocampus (rat)	Electroconvulsive Shock Therapy (10 days), Ketamine (single dose) and Fluoxetine (21 days of treatment)	Electroconvulsive shock therapy and ketamine modulated the expression of miR-598-5p in stressed animals	O’Connor et al. (2013)
miR-16	Hippocampus (mouse)	Fluoxetine (3 days of treatment)	Fluoxetine reduced miR-16 expression in the hippocampus	Launay et al. (2011)
miR-16	Raphe nuclei (mouse)	Fluoxetine (3 days of treatment)	Fluoxetine increased miR-16 levels in serotonergic raphe nuclei	Baudry et al. (2010)

### Human studies

Despite the efforts in the identification of predictors for antidepressant response and the growing evidence of a possible role of miRNAs, only a few studies have identified miRNAs as possible predictors of the antidepressant response. In Table 4, we presented all the clinical studies focused on the antidepressant drug response and miRNAs’ modulation in depressed patients. To identify the mentioned studies, we searched in PubMed using the keywords: “miRNAs”, “antidepressants”, “antidepressant response”, and “biomarker”. We found 19 relevant studies, and in the current paragraph, we will focus on the most investigated miRNAs: miR-1202, miR-16, and miR-124.

MiR-1202, a brain-specific miRNA, regulates the glutamate metabotropic receptor 4 (GRM4) that is involved in the glutamatergic, dopaminergic, GABAergic, and serotoninergic transmission (Pilc et al. 2008). GRM4 regulates both glutamatergic and non-glutamatergic transmission thanks to its pre- and post-synaptic location into neurons, which regulates the release of different neurotransmitters (Bradley et al. 1996; Cartmell and Schoepp 2000). Due to its involvement in several biological processes which have been associated with both depression development as well as drug response, the miR-1202 has been proposed as a novel potential target for the development of new antidepressant compounds (Lopez et al. 2014).

In this context, Lopez and colleagues showed reduced miR-1202 levels in post-mortem brains, specifically in the prefrontal cortex of depressed patients without a history of antidepressant treatment as compared to depressed patients in treatment with antidepressant drugs. The role of miR-1202 in the mechanisms of action of antidepressant drugs has been also investigated in neural precursor cells (NPCs), which have been treated with citalopram (SSRI) and imipramine (TCA) both acutely (24 h) and chronically (15 days). Only the chronic treatment showed an up-regulation of miR-1202 expression levels, followed by a reduction of GRM4 mRNA (Lopez et al. 2014). Furthermore, Lopez and colleagues investigated the regulation of miR-1202 in relation with citalopram treatment. Interestingly, patients who responded to the treatment showed lower baseline miR-1202 plasma levels as compared to both controls and to non-responder patients, whereas after 8 weeks of treatment, the miR-1202 expression levels in responders reached a peak, exceeding the levels observed in controls (Lopez et al. 2014), suggesting a possible role of this miRNA as a biomarker for antidepressant response. Other two independent studies reported the potential role of baseline miR-1202 levels in predicting treatment response to 8 weeks with escitalopram (SSRI) and desvenlafaxine (SNRI) treatment in depressed patients (Fiori et al. 2017), and they suggested that miR-1202 levels can predict the non-responsiveness to antidepressant treatment with a sensitivity of 91,7% and a specificity of 57,7%. Moreover, in line with the results of Lopez and colleagues, they also reported that 8 weeks of antidepressant treatment increased miR-1202 expression levels as compared to baseline (Fiori et al. 2017).

MiR-16 is a miRNA that has been widely implicated in the pathophysiology of depression; for examples, Baudry and colleagues demonstrated that the serotonin transporter (SERT) expression, a well-known vulnerability gene for depression, is modulated by miR-16 in 1C11 neuroectodermal cell line (Baudry et al. 2010). In particular, an over-expression of miR-16 resulted in a decrease in SERT protein levels as the inhibition of the miRNA is associated with an increase in SERT protein expression. Accordingly, the work of Song and collaborators showed a down-regulation of miR-16 expression levels in CSF of drug-free depressed patients compared to controls along with reduced CSF serotonin levels, since a down-regulation of miR-16 is associated with an up-regulation of SERT, and as a consequence, this leads to an increase in the serotonin reuptake (Song et al. 2015). Based on these findings, researches tried to evaluate a possible utility of miR-16 as biomarker associated with the mechanisms underlying the effects of for antidepressant drugs. For example, Lin and colleagues showed increased miR-16 levels in depressed patients after 4 weeks of SSRI treatment compared to baseline; however, no changes in miR-16 levels were found in the group of patients treated with SNRI, suggesting that antidepressant drugs with different mechanisms of action might exert their action via involvement of specific and different sets of miRNAs (Lin et al. 2018).

The serotonin receptor-1a transcripts and the SERT are also targeted by miR-135. Issler and colleagues showed increased blood miR-135 levels in depressed subjects undergoing cognitive behavioural therapy (CBT), but not in depressed patients treated with escitalopram for 12 weeks, suggesting that miR-135a in human blood might be a possible biomarker for the antidepressant drug effect; however, larger cohorts are required to investigate its possible clinical involvement (Issler et al. 2014).

MiR-124 is known to regulate cell growth, proliferation, and apoptosis in the brain (Cheng et al. 2009), and a significantly higher expression of miR-124 in peripheral blood mononuclear cells (PBMCs) has been reported in depressed patients at baseline before escitalopram treatment; moreover, miRNA levels sharply decreased after 8 weeks of therapy, especially in responders, suggesting a role of this miRNA as biomarker for treatment efficacy (He et al. 2016). These results have been also validated in other cohorts where miR-124 levels have been found reduced after 4 weeks of treatment with the traditional Chinese antidepressant medicine Chaihu-Shigau-San; also, this miRNA decrease paralleled an up-regulation of the mRNA and of protein levels of mitogen-activated protein kinase 14 (MAPK14) and glutamate receptor subunit 3 (Gria3), which are miR-124 predicted target genes (Liu et al. 2018).

### Animal models

To discuss papers describing miRNAs modulated by antidepressant drugs and identify miRNAs as potential biomarkers for antidepressant response, we performed a search in PubMed using the following keywords: “animal models”, “rats”, “mouse” “miRNAs”, “antidepressants”, “antidepressant response”, and “biomarkers”, and we considered only studies describing the role of miRNAs in the mechanisms underlying the effects of antidepressant drugs. We found 15 relevant studies (Table 5), and in this paragraph, we have decided to focus on miR-16 and miR-124 only, as they have been investigated in at least two independent studies.

MiR-16 has been suggested to be involved in the effect on hippocampal neurogenesis induced by SSRI treatment (Launay et al. 2011). Interestingly, Launay and colleagues showed that a 3-day stereotaxic injection in the raphe nuclei of mice with the SSRI fluoxetine induced a down-regulation of miR-16 levels in the hippocampus, an effect that was significantly correlated with an increase in SERT expression. Conversely, the direct injection of anti-miR-16 in the hippocampus increased the levels of SERT, similarly to fluoxetine injection in the raphe nuclei. These findings suggest that the fluoxetine-mediated down-regulation of miR-16 in the hippocampus has an antidepressant activity (Launay et al. 2011).

However, the data are in contrast with the results of another study conducted on human cohorts that we have already previously described (Song et al. 2015; Lin et al. 2018). Particular attention to miR-16 activity has been paid also by Baudry et al. (2010) as they measured miR-16 expression levels in the raphe nuclei of mice after a 3-day infusion with fluoxetine, reporting an increase in the miR-16 levels. The ability of fluoxetine to induce an up-regulation of this miRNA is probably due to the activity of a pre/pri-miR-16, whose levels are inversely correlated to those of the mature miRNA (Baudry et al. 2010). On the other hand, a 20-day infusion of fluoxetine reduced miR-16 expression both in mice hippocampus and raphe (Baudry et al. 2010; Launay et al. 2011).

MiR-124a has been found modulated by antidepressant treatment duloxetine: adult mice were exposed to a set of chronic unpredictable mild stressors for 4 weeks and then received the SNRI duloxetine for 3 weeks, and an up-regulation of miR-124a and miR-134 and a down-regulation of miR-132 and miR-18 were observed in the hippocampus of mice exposed to stress. Moreover, after the administration of duloxetine, the increased levels of both miR-124a and miR-134 which occurred after stress significantly dropped, suggesting a direct modulation of these miRNAs mediated by the SNRI drug (Pan and Liu 2015). The modulation of miR-124a by antidepressant drugs was investigated also by Higuchi and colleagues in a group of mice exposed to chronic ultra-mild stress. The animals were exposed to the stressful paradigm for 6 weeks and then received the tricyclic antidepressant drug imipramine for the last 3 weeks of the paradigm. The levels of pri/pre miR-124a and the mature miR-124a were down-regulated in the hippocampus of stressed mice, and this effect was blocked by the administration of imipramine, both for pri/pre and the mature miR-124a, suggesting that the modulation of miR-124a is mediated by imipramine (Higuchi et al. 2016).

To conclude, miRNAs have been shown to be widely modulated by both pharmacological and not-pharmacological antidepressant treatments in clinical as well as pre-clinical studies. The identification of miRNAs, whose levels in depressed patients can be predictive of the treatment response, might represent a useful tool to be used in the clinical practice in helping the clinician to early determine the future response to treatment. Moreover, these miRNAs can represent novel target for the development of new antidepressant drugs.

## miR-based therapeutics

RNA interference (RNAi) is a regulatory approach based on the use of small double-stranded RNA (dsRNA) molecules as triggers to direct homology-dependent control of gene activity (Almeida and Allshire 2005). The RNAi can be artificially induced by introducing a small double-stranded fragment of RNA, which corresponds to a particular mRNA into a cell. The RISC complex within the cell recognizes this double-stranded RNA fragment and uses the guide strand to bind and destroy its corresponding cellular mRNA target, inhibiting the translation of the encoded protein. Indeed, as a dysregulation of miRNAs targeting specific genes could play a key role in the development of psychiatric disorders, a specific modulation of miRNAs expression might represent an innovative therapeutic tool to work on the pathological course of the disease. Artificial RNAi could thus represent an example of inhibition of the translation of specific proteins which play active roles in the development of psychiatric diseases (Castanotto and Rossi 2009).

Similarly, the inhibition of certain miRNAs, named miRNAs mimics, which contain the same sequences of the endogenous miRNA of interest (Matsukura et al. 2016), represents another technique that is increasingly being used to over-express a specific miRNA and, therefore, to examine the biological effects associated with such over-expression.

However, to date, several drawbacks and challenges associated with miRNAs silencing and gain-of-function experiments in the research of psychiatric disorders are still uncertain. The first RNA-based therapeutic strategies had a very low bioavailability due to the potential degradation of the oligonucleotides by RNase in the serum or in endocytic compartment of cells (Rupaimoole and Slack 2017). Moreover, the difficulties of reaching the target site by RNA drugs are noteworthy due to complexity in targeting the oligonucleotides to specific tissues and body fluids. Due to all these issues, the first published studies were mostly unsuccessful raising the need to improve the methodology.

To avoid issues related to the bioavailability, different strategies have been then applied: (1) modifying the chemistry of oligonucleotides by altering the nucleotides backbone or adding phosphonothioate groups; and (2) developing delivery systems able to enclose miRNAs. The former approach has been the most widespread, whereas, only more recently, more efforts have been made to develop non-toxic and safe delivery systems.

Up to date, only one RNAi-based therapy has been approved by Food and Drug Administration (FDA) and European Medicines Agency (EMA) on August 2018. The new drug is called patisiran (Onpattro) and it aims to treat polyneuropathy caused by a rare and frequently fatal disease called hereditary transthyretin-mediated amyloidosis (hATTR). Patisiran is the first RNAi drug approved by FDA after more than 20 years of research and it marks the arrival of a game-changing new class of therapeutics. Furthermore, RNAi-based therapy is currently in the pre-clinical phase or is entering clinical trials; these emerging drugs have been engineered to treat mainly cancers and hepatitis C virus-related disorders, as well as cardiovascular diseases (Chakraborty et al. 2017). Unfortunately, no miR-based therapeutics for the treatment of psychiatric diseases have reached the pre-clinical stage during the past years, showing the urgent need to increase the efforts of identifying miRNAs targeting molecules known to be involved in the pathogenesis of specific psychiatric disorders.

## Conclusions

According to the World Health Association, depression is becoming the most debilitating disease worldwide with a social and economic burden that is constantly increasing. Exposures to stress early in life, such as childhood trauma events, represent one of the most important risk factors for the development of depression, as it targets several biological systems, such as the HPA axis, which in turn contribute to the pathogenesis of the disorder.

It is important to note that not all the exposed individuals develop a psychopathology later in life, as some of them develop coping strategies that render them more resilient. During the last decades, researchers have focused their attention on the identification of mechanisms associated with stress vulnerability and stress resilience as this could allow the identification of novel pathways, mechanisms, and molecules that if properly targeted may reduce the vulnerability or may potentiate the resilience. Among the candidate mechanisms, researchers have paid attention on epigenetic modifications, including miRNAs.

Our revision of the literature supports the hypothesis of the involvement of miRNAs in the mechanisms underlying stress response and in those associated with the development of depression; moreover, miRNAs could be particularly interesting as potential peripheral biomarkers for the identification of vulnerable or resilient individuals. MiRNAs have been also found to be involved in the mechanisms associated with treatment response, and thus, a better understanding of their role may reveal novel potential targets for the development of novel and more efficient therapies. Finally, miRNA-based therapeutics, including RNAi and over-expressing miRNAs approaches, have been proposed as therapeutic tools to directly target genes involved in the pathogenesis of depression. However, authorities (FDA or EMA) have not yet approved these drugs, and more studies are required to better implement these tools and to employ miRNAs in the clinical settings.
